# Do Commercial Airline Policies for Passengers With Obesity Carry Enough Weight?

**DOI:** 10.1002/osp4.70132

**Published:** 2026-03-11

**Authors:** Tom McGovern, Francis M. Finucane, Gerard T. Flaherty

**Affiliations:** ^1^ School of Medicine, College of Medicine, Nursing and Health Sciences University of Galway Galway UK; ^2^ National Institute for Prevention and Cardiovascular Health Galway UK; ^3^ Bariatric Medicine Service, Centre for Diabetes, Endocrinology and Metabolism Galway University Hospitals Galway UK; ^4^ Cúram University of Galway Galway UK

## Abstract

**Introduction:**

The travel industry has a responsibility to accommodate the needs of all its customers, including those with obesity. It is not known to what extent airlines communicate accessibility policies to passengers with obesity. We sought to assess the adequacy and content of information provided by international commercial airlines regarding the carriage of passengers with obesity.

**Methods:**

A descriptive cross‐sectional analysis was conducted of policies relating to passengers with obesity that were available on publicly accessible websites of the 50 busiest global commercial airlines. Variables of interest included fleet description, passenger weight‐related terminology, seating information, use of seatbelt extenders, special assistance information, use of artificial intelligence chatbots for customer service, and availability of customer information in the English language.

**Results:**

A diverse range of terms was used to describe travelers with obesity, with “customers requiring extra personal space” or “an extra seat for personal comfort” being the most frequently used descriptors. Seating information relating to larger passengers was provided by 86% (*n* = 43) of airlines. Passenger guidance on the use of seatbelt extenders was available on 70% (*n* = 35) of airline websites. Special assistance information was provided by all airlines.

**Conclusion:**

While leading commercial airlines provide obesity policy information on their websites, there is considerable variation between carriers in the degree of information provided. There is a need for more transparent and standardized accessibility information for airline passengers with obesity.

## 1 Introduction

Obesity is highly prevalent [1] and people living with this chronic disease continue to face high levels of discrimination and prejudice in many aspects of daily life. A previous study described the unique vulnerability of international travelers with obesity [2], and the significant challenges they face [3]. Some of the greatest concerns for travelers relate to the requirement to purchase extra seats on flights, navigating airport terminals, boarding aircraft, and accessing airplane toilets [3]. Perceived prejudice from passengers without obesity and members of the travel industry contributed to a general reluctance among people with severe obesity to travel overseas.

The mental health benefits of international travel have received greater attention since COVID‐related travel restrictions were lifted [4]. A recent commentary pointed to the therapeutic value of travel for people living with a chronic illness and their family carers, with reductions in psychological stress, detachment from daily routine, and opportunities for personal growth among its greatest potential benefits [4]. The travel industry, including commercial airlines, has a responsibility to cater for the needs of special groups of travelers, including those with obesity. An estimated 5 billion passengers were carried by the world's commercial airlines in 2024 [5]. There has been very limited research exploring the interface between travelers with additional needs and international airlines. One study of wheelchair users with spinal cord injury revealed physical barriers to airplane accessibility in this population. Inadequately trained staff and a transfer of responsibility to the passenger with a disability compromised the passengers' air travel experience [6]. It is not known to what extent airlines currently meet the specific needs of passengers with obesity and how this information is communicated through their websites. The present study sought to assess the information provided on their websites by leading international commercial airlines regarding the carriage of travelers with obesity.

## 2 Methods

A cross‐sectional descriptive content analysis was conducted of policies relating to passengers with obesity that were available on the publicly accessible websites of the top 50 commercial airlines globally. Airlines were included if they ranked in the top 50 airlines by daily flight departure volumes (https://www.flightsfrom.com/top‐100‐airlines) and were not a subsidiary company of a parent airline or an aircraft leasing company. Websites were accessed and data extracted between February and June 2025. Variables of interest were developed by consensus and were based on the findings of previous qualitative research among travelers with obesity [3]. They included the number of daily flights, fleet description, passenger weight‐related terminology used, seating information, use of seatbelt extenders, special assistance information, use of artificial intelligence (AI) chatbots for customer service support, and availability of customer information in the English language. Any additional information derived from the use of the chatbot query facility was recorded. Embedded website translation tools were used where information was published in languages other than English.

An initial search sought information specifically related to passengers with obesity. Web pages with the titles “Special Assistance,” “Our Fleet,” “At the Airport,” “In Flight Experience,” and “Seating Options” were also examined. The layout of these pages varied by airline website. The website frequently answered questions section and any available chatbot function were also used to help navigate the website and access relevant information using specific prompts. No standardized keywords were used to search the websites given the variability of website layout and content.

Website data were manually extracted independently by two researchers (T.M. and G.T.F.) and entered in a Microsoft Excel spreadsheet. An aggregate airline score with a maximum total of nine was computed based on the presence or absence of the following website information relating to travel with obesity: information relating to passengers with obesity; relevant seating information; seatbelt extenders; mobility aids; special assistance; aircraft fleet dimensions; availability in English; chatbot or search tools; non‐stigmatizing descriptions of passengers with obesity. Data were analyzed descriptively using frequencies and proportions. As the study did not involve human subjects, no ethics board review was required.

## 3 Results

Relevant information was accessible on the websites of each of the 50 airlines with the highest daily flight volumes (Supporting Information S1). The combined daily flight volume was 49,661. The mean daily number of flights was 993 (standard deviation = 939.40), with the highest number of flights completed by the US‐based airlines American Airlines (*n* = 4710), United Airlines (*n* = 3593), and Delta Airlines (*n* = 3461). While all the airlines had English websites, some content was published only in the national language of the parent airline (10%, *n* = 5). A diverse range of euphemistic phrases (*n* = 15) was used to describe travelers with obesity (Table 1), with “customers requiring extra personal space” or “an extra seat for personal comfort” being the most frequently used descriptors. The potentially stigmatizing phrase “over‐sized passengers” was applied by a single airline. Approximately 8% (*n* = 3) of airlines which made any reference to passengers of larger size used the terms “obese” or “obesity,” and no airline provided a workable definition by reference to body mass index or other parameters.

**TABLE 1:** Obesity‐related terminology used on airline websites.

Terminology referring to air passengers with obesity	Use by airlines (*n*)
Extra seat for personal comfort	6
Customers requiring extra personal space	6
Customers/passengers/guests of size	5
Passengers who require an extended seat belt	4
Of a large build/larger/larger‐bodied/bigger passengers	4
People with obesity	2
Unable to be seated in a single seat due to physical build/size	2
Requiring more than one seat	1
Cannot sit comfortably in their seat	1
Special passengers/persons with disabilities	1
Extra seat for medical reasons	1
Customers wishing to have two or more seats	1
Passengers who cannot fit into an economy class seat	1
Obese passengers	1
Size does not allow for quick action in the event of an emergency	1
Over‐sized passengers	1

Seating information relating to passengers of larger size was provided by 86% (*n* = 43) of airlines. Passenger guidance on the use of seatbelt extenders was available on 70% (*n* = 35) of airline websites. Special assistance information was provided by all airlines (*n* = 50). The airline fleet dimensions and seating policies were described in 96% (*n* = 48) of cases (Table 2). A minority of airline websites (26%, *n* = 13) used AI‐based chatbot facilities to communicate with their customers. Of all airlines, 36% (*n* = 18) advised passengers to request a seatbelt extender from a flight attendant, 2% (*n* = 1) from the departure gate, and 24% (*n* = 12) in advance of travel. Twenty‐four percent (*n* = 12) of airlines stipulated that passengers were not permitted to use their own extender. A single airline provided the exact dimensions of its seat belts for all its airfare classes.

**TABLE 2:** Airline seating specifications and special assistance policies.

Airline website information item	Provision by airlines (*n*, %)
Seating information	
Seat width	28 (56)
Seat pitch or legroom	31 (62)
Aisle width	1 (2)
Cabin door size	2 (4)
Movable armrests	35 (70)
Accessible lavatories^a^	26 (52)
Presence of toilet grab bar	9 (18)
Larger/higher class seat advised	12 (24)
Offer option to book extra seat	41 (82)
Refunds/provides free extra seat if extra space needed^b^	2 (4)
Discount on extra seat	6 (12)
Refunds seat if flight not full	3 (6)
Advised to check for serviceable seat on boarding	7 (14)
Early boarding for those needing extra space	16 (32)
Purchase extra seat on day of travel at extra cost	5 (10)
Passenger denied boarding if unable to fit in seat	6 (12)
Eligibility to occupy emergency exit row	23 (46)
Exclusion from access to emergency exit row	24 (48)
Special assistance policy	
Airport terminal assistance	50 (100)
Assistance navigating airplane aisle	35 (70)
Assistance getting into and out of seat	10 (20)
Storing luggage in overhead compartment	20 (40)
Opening food or drink containers	13 (26)
Assistance with eating	1 (2)
No assistance with passenger's medication provided	41 (82)
No assistance with using bathroom provided	39 (78)
Preboarding facility if mobility restrictions	30 (60)
Assistance with boarding	48 (96)
Assistance with disembarkation	41 (82)
Advised booking traveling companion for assistance	48 (96)
Discounted ticket for assisting traveling companion	7 (14)
May request trained medical personnel to assist	5 (10)
Own collapsible wheelchair can be brought on board	32 (64)
Onboard wheelchair provided^c^	39 (78)
Passenger must request onboard wheelchair in advance	19 (38)

^a^ One airline reminded passengers that airplane toilets are generally cramped and aisles narrower than on other forms of transport. Another airline gave exact toilet dimensions.

^b^ One airline specified that a 20% discount was available if an extra seat was booked in advance of travel.

^c^ One airline provided detailed dimensions for the onboard wheelchair.

Special assistance policies were published by all 50 airlines and are presented in Table 2. The airlines with the highest aggregate score (total of nine points) for website information relating to passengers with obesity were as follows: Air India, Air New Zealand, Al Nippon Airways, Delta, IndiGo, Jetstar, Lufthansa, Pegasus, and VietJet Air.

## 4 Discussion

With a growing emphasis on the mental health benefits of travel [7], it is important that barriers to safe and fulfilling travel are minimized. Previous studies have highlighted the general issues facing international travelers with varying degrees of disability [8], including the dehumanizing experiences reported by some disabled air passengers [9]. International airlines have faced criticism for introducing voluntary passenger weighing systems to collect data for flight balance calculations [10], or in some cases proposing that heavier passengers pay more for their tickets based on body weight [11]. While the main responsibility of the commercial aviation sector is to ensure passenger safety, there is also an obligation on airlines to accommodate diverse passengers' needs and promote their wellbeing, comfort and dignity. The policies of airlines concerning individuals with obesity thus warrant critical examination.

Airlines typically develop policies that focus on the provision of additional seating and specific boarding procedures for passengers with obesity. The present study demonstrates the efforts made by airlines to address the needs of this traveler demographic, but it also highlights the inconsistency in these policies across leading carriers, leading to concerns about fairness and accessibility. A core issue emerging from this analysis of airline website information for passengers with obesity is the widely varying nomenclature used to describe such passengers, and the lack of use of a formal definition of obesity. There is a lack of industry standards in this domain. It has been accepted that people with obesity need special consideration in other aspects of life, such as in the design of hospitals [12], furniture, and automobile manufacture. Airlines should adopt universal policies in this field, so that the same standards of societal and organizational responsibility that have already been established in other sectors can be applied to aviation.

Airlines’ legal responsibilities toward passengers with obesity are primarily governed by accessibility and disability regulations, such as European Regulation (EC) No 1107/2006 on the rights of persons with reduced mobility [13]. These regulations require airlines to have a duty of care, including providing information, considering safety needs, and justifying any refusals of service. Airlines are not legally obligated to provide larger custom seats, but they must have clear justified policies for passengers whose size prevents them from fitting within standard seating, often by requiring the purchase of an additional seat to ensure their safety and the safety of others [14].

While most airlines offer passengers the option to purchase an additional seat if they encroach upon the space of adjacent passengers, reimbursement policies varied widely between airlines in this analysis. Only 12% of carriers offered a discount on the extra seat and 6% communicated a policy about refunding the cost of the seat when a flight is not fully booked. Apart from the financial hardship involved in purchasing an extra seat, which may or not be discounted, extra seat policies may inadvertently stigmatize people with obesity and generate feelings of humiliation or perceived prejudice.

Airlines must balance the need for passenger comfort with operational considerations around safety, weight distribution, and cabin space. Furthermore, the United States Federal Aviation Administration, in cooperation with the International Civil Aviation Organization, mandates safety standards that require airlines to ensure that all passengers can be safely evacuated in an emergency [15]. Such regulations can be difficult to harmonize with seating policies aimed at greater inclusivity.

This study may help to identify areas for improvement in the travel experience of air passengers with obesity. We recommend that legislators, policy developers, and regulators strive for objectivity and clarity on this issue, which should translate into improved carrier policies, passenger communication, and staff training. Aviation regulators could consider the implementation of more standardized policies that accommodate passengers of all sizes while safeguarding passenger safety and comfort. A more consistent use of terminology which does not stigmatize but communicates airline policies with clarity and without the use of euphemisms, would be desirable. Aircraft design could incorporate a greater variety of seat configurations in the economy class cabin with wider aisle access to lavatories with sliding doors, and seatbelt extenders discreetly provided in the seat pockets. The application of a more inclusive pricing structure would reduce financial barriers to air travel in this population. Training of flight crew to sensitively handle the preboarding, luggage stowing, and seating of passengers with obesity would help to mitigate their discomfort and stigmatization.

Although this is the first study to specifically explore airline website obesity policies, it is limited by its restriction to the top 50 airline carriers globally and to airlines whose websites are available in the English language. It is estimated that this group of airlines accounts for approximately half of the world's approximately 100,000 daily commercial flight take‐offs. Exemplary policies operated by smaller airlines may therefore have been overlooked. Individual airlines were not contacted where online policy information was absent or lacking, and it is acknowledged that airlines will often endeavor to apply discretion in the case of individual travelers with specific or unique needs. This study was not designed to capture airlines' individual efforts to accommodate passengers with obesity but rather the quality of their communication of relevant policy information to this passenger cohort. There is likely to be significant variation in passenger experience of individual airlines depending on other personal health‐related factors, such as grade of obesity, presence of medical comorbidities such as osteoarthritis and obstructive sleep apnea in passengers with higher grades of obesity, and passenger mobility. This study was not designed to evaluate the implementation of airline obesity policies or airline compliance with disability legislation.

Further research should employ social media listening strategies to examine the lived experiences of airline passengers with obesity on public discussion forums, with a focus on how airline accessibility impacts their travel decisions, airline choices, and journey experience. Follow‐up studies should use a focus group methodology to explore passengers' experiences of navigating airline websites to search for relevant policy information. Implementation studies would be worthwhile based on the principles of website communication emerging from this study.

## 5 Conclusion

While leading commercial airlines endeavor to provide policy information on their websites that is relevant to passengers with obesity, this information is often insufficient or incomplete and there is considerable variation between carriers in the information provided. There is a need for greater standardization in the provision of accessibility information to airline passengers with obesity. To enhance customer inclusivity and ensure equity of access, airlines should strive to improve the online communication of their obesity policies, notwithstanding the onus on airlines for safe carriage and protection of the interests of fellow passengers without obesity.

## Author Contributions

**Tom McGovern:** methodology, data analysis, data interpretation, and editing of the draft manuscript. **Francis M. Finucane:** conceptualization, data interpretation, and editing of the draft manuscript. **Gerard T. Flaherty:** conceptualization, methodology, data interpretation, supervision, and preparation of the draft manuscript.

## Funding

The authors have nothing to report.

## Conflicts of Interest

F.M.F. has received payment from the University of Michigan for membership in the Data Safety Monitoring Board of the LEAP and LEGEND randomized controlled trials, and from the Danish Diabetes Academy for reviewing grant applications. He is an investigator (unpaid) on two Novo Nordisk‐sponsored clinical trials, REDEFINE 2 and REDEFINE 3. There are no other personal or financial conflicts of interest to disclose.

## Supporting information


Supporting Information S1

## Data Availability

The data that support the findings of this study are available from the corresponding author upon reasonable request.

## References

[1] GBD 2021 Adult BMI Collaborators , “Global, Regional, and National Prevalence of Adult Overweight and Obesity, 1990–2021, With Forecasts to 2050: A Forecasting Study for the Global Burden of Disease Study 2021,” Lancet 405, no. 10481 (March 2025): 813–838, 10.1016/S0140-6736(25)00355-1. 40049186 PMC11920007

[2] M. V. Mozo , F. M. Finucane , and G. T. Flaherty , “Health Challenges of International Travel for Obese Patients,” Journal of Travel Medicine 24, no. 6 (2017): 1–4, 10.1093/jtm/tax065. 29088479

[3] G. T. Flaherty , R. Geoghegan , I. G. Brown , and F. M. Finucane , “Severe Obesity as a Barrier to International Travel: A Qualitative Analysis,” Journal of Travel Medicine 26, no. 3 (2019): taz018, 10.1093/jtm/taz018. 30855079

[4] G. T. Flaherty , R. Steffen , and K. Leder , “Towards Travel Therapy: Addressing the Health Benefits of International Travel,” Journal of Travel Medicine 32, no. 3 (2025): taae091, 10.1093/jtm/taae091. 38959849

[5] Air Transport Action Group , “Facts & Figures,” (2023), https://atag.org/facts‐figures.

[6] J. L. Pfeiffer , W. Bower , and P. Rumrill , “Investigating the Challenges of Air Travel in the United States: A Qualitative Study of the Lived Experiences of Wheelchair Users With Spinal Cord Injury or Disorder,” Spinal Cord Series and Cases 10, no. 1 (April 2024): 25, 10.1038/s41394-024-00641-6. 38643214 PMC11032379

[7] G. Flaherty , S. Y. Chai , and B. Hallahan , “To Travel Is to Live: Embracing the Emerging Field of Travel Psychiatry,” BJPsych Bulletin 45, no. 3 (2021): 167–170, 10.1192/bjb.2020.32. 32279684 PMC9027445

[8] I. Bauer , “When Travel Is a Challenge: Travel Medicine and the ‘Dis‐Abled’ Traveller,” Travel Medicine and Infectious Disease 22 (2018): 66–72, 10.1016/j.tmaid.2018.02.001. 29454050

[9] D. Gotti , E. Morales , F. Routhier , J. Riendeau , and A. H. Hassen , “Dehumanizing Air Travel: A Scoping Review on Accessibility and Inclusion of People With Disabilities in International Airports,” Frontiers in Rehabilitation Sciences 5 (2024): 1305191, 10.3389/fresc.2024.1305191. 39329061 PMC11425591

[10] British Broadcasting Corporation , “Finnair Sparks Controversy by Weighing Its Passengers,” (2024), https://www.bbc.com/travel/article/20240221‐finnair‐airlines‐weighing‐passengers‐controversy.

[11] British Broadcasting Corporation , “Samoa Air Boss Defends Charging Passengers by Weight,” (2013), https://www.bbc.com/news/world‐22001256.

[12] D. Wignall , “Design as a Critical Tool in Bariatric Patient Care,” Journal of Diabetes Science and Technology 2 (2008): 263–267, 10.1177/193229680800200216. 19885353 PMC2771495

[13] European Union , “Regulation (EC) No 1107/2006 of the European Parliament and of the Council of 5 July 2006 Concerning the Rights of Disabled Persons and Persons With Reduced Mobility When Travelling by Air,” (2006), https://eur‐lex.europa.eu/legal‐content/EN/TXT/?uri=CELEX%3A32006R1107.

[14] European Aviation Safety Agency , “Carriage of Special Categories of Passengers (SCPs),” (2014), https://www.easa.europa.eu.

[15] Federal Aviation Administration , “Safety Information,” (2022), https://www.faa.gov/travelers/fly_safe/information.

