# Supplementary material for: Do Commercial Airline Policies for Passengers With Obesity Carry Enough Weight?
Source: Obes Sci Pract. 2026 Mar 11;12(2):e70132. doi: 10.1002/osp4.70132 (PMC12977105; doi:10.1002/osp4.70132)
Supplement: Supplementary file 1 — Supporting Information S1 [file OSP4-12-e70132-s001.docx]

**Supplementary File.** Top 50 global airlines according to number of daily flights

| **Airline** | **Daily Number of Flights** |
| --- | --- |
| American Airlines  United Airlines  Delta Air Lines  China Eastern Airlines  Southwest Airlines  IndiGo  China Southern Airlines  Ryanair  Air China  ANA  LATAM Airlines  Air Canada  Turkish Airlines  JAL  Shenzhen Airlines  Hainan Airlines  easyJet  Azul  AVIANCA  Air India  Alaska Airlines  Xiamen Airlines  Aeroflot  Lufthansa  Qantas  KLM  JetBlue Airways  Shandong Airlines  Air France  Spirit Airlines  Sichuan Airlines  British Airways  AirAsia  Iberia  Qatar Airways  Pegasus  Saudia  Emirates  Spring Airlines  Volaris  Gol  Ethiopian Airlines  VietJet Air  Air India Express  VivaAerobus  Jetstar  Vueling  Air New Zealand  Cebu Pacific Air  Virgin Australia | 4710  3593  3461  2500  2464  2229  2215  1962  1670  1179  1171  925  876  832  803  796  761  756  737  736  721  642  641  637  637  609  609  600  580  577  567  558  531  523  485  480  464  462  456  442  435  430  428  416  416  414  392  382  382  369 |

Source: <https://www.flightsfrom.com/top-100-airlines>
